# Linking Developmental HRM to Organizational Dehumanization Among Nurses: Work Meaningfulness as a Mediator and Mastery Climate as a Moderator

**DOI:** 10.1155/jonm/8009052

**Published:** 2025-11-29

**Authors:** Yao Song, Yang Liu, Shanshan Li

**Affiliations:** ^1^School of Economics and Management, Beijing University of Chemical Technology, Beijing, China; ^2^Business School, Central University of Finance and Economics, Beijing, China; ^3^Tengzhou Central People's Hospital, Tengzhou, Shandong, China

**Keywords:** developmental human resource management, mastery climate, organizational dehumanization, work meaningfulness

## Abstract

**Background:**

Nurses may experience organizational dehumanization, perceiving themselves as mere tools to achieve institutional goals rather than as valued healthcare professionals. Such perceptions not only harm their well-being and work engagement but also threaten the quality and sustainability of healthcare delivery. While prior research has primarily focused on the negative consequences of organizational dehumanization, limited attention has been given to identifying effective organizational strategies to alleviate this issue. Drawing on self-determination theory, this study explores whether, how, and when developmental human resource management (DHRM) practices can mitigate nurses' organizational dehumanization.

**Methods:**

We conducted a three-wave time-lagged study with 311 nurses across multiple hospitals. Hypotheses were tested using structural equation modeling.

**Results:**

DHRM was positively related to nurses' work meaningfulness, which in turn was negatively associated with organizational dehumanization. Work meaningfulness mediated the relationship between DHRM and dehumanization. In addition, mastery climate strengthened the positive effect of DHRM on work meaningfulness, thereby amplifying its indirect association with reduced dehumanization.

**Conclusions:**

This study highlights the value of implementing comprehensive DHRM practices in healthcare settings to enhance nurses' experience of meaningful work and alleviate organizational dehumanization. Additionally, fostering a mastery-oriented climate within hospitals can further strengthen the positive influence of DHRM.

## 1. Introduction

“Some days, I feel like just another cog in the hospital machine—replaceable, unnoticed, and running endlessly without purpose.”

—According to one nurse participant.

The healthcare industry is regarded as a sacred field, dedicated to the well-being and health of individuals [1]. Central to this mission are nurses, who seek purpose and fulfillment in their roles, driven by the desire to care for others and make a meaningful impact [2]. However, as healthcare demands rise, nurses face growing challenges, including workforce shortages and mounting work pressures [3]. Reports from the International Council of Nurses highlight that nurses frequently endure long working hours, irregular shifts, and heavy patient loads, under conditions of insufficient staffing. Alongside physical exhaustion, they also shoulder intense emotional labor, expected to deliver compassionate care even when feeling drained. In such taxing environments, the pursuit of meaningful work is compromised, leaving nurses struggling to align their daily tasks with their deeper professional values. Consequently, many begin to feel dehumanized [3, 4], perceiving themselves as mere cogs in an impersonal machine—interchangeable tools serving organizational goals rather than individuals with unique contributions [5, 6]. Such organizational dehumanization erodes intrinsic motivation and undermines the fulfillment derived from patient care [7]. Research shows that it leads to harmful outcomes, including heightened stress, deviant behaviors, and diminished engagement [3, 4]. Ultimately, this increasing sense of dehumanization can harm nurses' well-being and performance and may also put the long-term stability of the healthcare system at risk. These challenges point to the need for strategies that can help nurses find greater meaning in their work and ease experiences of organizational dehumanization.

Despite growing interest in its antecedents, the literature has not yet provided a clear account of how organizations can respond to or mitigate such perceptions. The existing literature has primarily focused on the detrimental effects of organizational dehumanization [3, 4, 7, 8], with a more recent stream of research beginning to examine its antecedents [9–11]. However, relatively little attention has been paid to identifying organizational practices that can help alleviate or buffer against these negative perceptions. This is a key challenge, as addressing organizational dehumanization is crucial not only for improving nurses' performance but also for ensuring the long-term development and success of healthcare institutions [12, 13]. Unfortunately, there is a significant lack of research on practical interventions in this area within hospital settings. This gap highlights the pressing need for further research focused on effective strategies to combat organizational dehumanization—research that could provide actionable insights for hospital administrators.

To address this issue, we focus on human resource management (HRM), recognizing that HR practices play a pivotal role in shaping nurses' work experiences and psychological well-being [14, 15]. Specifically, we focus on developmental HRM (DHRM)—a set of proactive practices, including career development, training opportunities, and constructive performance appraisal—designed to enhance employees' skills and motivation by addressing their growth and developmental needs [16, 17]. Unlike traditional HRM approaches that emphasize control or efficiency, DHRM emphasizes investment in employees' long-term development and empowerment. It signals to nurses that they are valued as individuals with potential, not merely as resources to be deployed. This is particularly important in the nursing context, where heavy workloads and emotional demands are associated with stress, disengagement, and even feelings of organizational dehumanization. By highlighting growth, support, and recognition, DHRM not only develops competencies but also strengthens nurses' sense of dignity, value, and purpose in their work.

Accordingly, we contend that DHRM provides not only structural resources for growth but also a crucial pathway through which nurses can buffer against organizational dehumanization. Drawing on self-determination theory (SDT), which emphasizes that fulfilling the basic psychological needs of autonomy, competence, and relatedness fosters intrinsic motivation [18], we argue that DHRM can cultivate a sense of work meaningfulness by supporting nurses' personal and professional growth. For instance, by providing opportunities for career advancement, skill development, and regular performance feedback, DHRM satisfies nurses' core psychological needs, thereby helping them rediscover meaning in their work [19]. In particular, we highlight work meaningfulness as the most relevant psychological mechanism in this process, as it mitigates feelings of organizational objectification and provides a more explicit and observable pathway than general needs fulfillment. This, in turn, strengthens their sense of work meaningfulness, fosters a deeper psychological connection to their professional roles, and ultimately mitigates feelings of organizational dehumanization.

**Figure 1:**
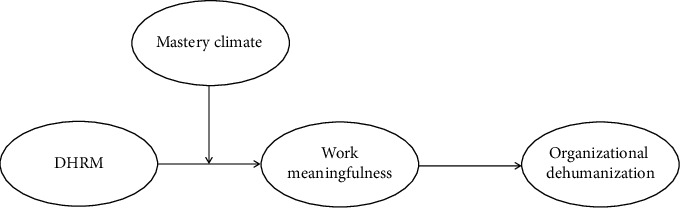
Theoretical model.

Furthermore, this study argues that effectively mitigating organizational dehumanization requires attention not only to formal HRM systems but also to the broader organizational climate. According to SDT, situational factors such as a mastery climate play a crucial role in fulfilling employees' psychological needs [20, 21]. A mastery climate—a motivational environment that emphasizes effort, collaboration, and skill development [22, 23]—acts as an amplifier of DHRM practices. While DHRM practices provide a structured framework for nurses' development, a mastery climate fosters the supportive atmosphere that makes these practices more effective. In a mastery-oriented climate, nurses are more likely to perceive DHRM as authentic support for their development, internalize the value of personal growth, and feel a stronger sense of work meaningfulness [24, 25]. Thus, mastery climate does not merely complement DHRM—it magnifies its positive effects, deepening nurses' sense of meaning at work and ultimately reducing experiences of organizational dehumanization. The proposed research model is shown in Figure 1.

This study offers three important contributions to nursing management and healthcare human resource practices. First, by examining how DHRM practices can alleviate organizational dehumanization, this study extends the nursing management literature with actionable strategies for creating more humane work environments. Prior research has mainly emphasized the detrimental effects of organizational dehumanization and, more recently, its antecedents [7–11], but these studies largely focus on risk factors rather than on practices that can buffer against or mitigate such perceptions. Second, by uncovering the mediating role of work meaningfulness, this study deepens the understanding of the psychological mechanisms through which organizational dehumanization can be reduced [12, 26]. In particular, it highlights how DHRM practices in healthcare settings can foster nurses' sense of purpose and professional growth, ultimately promoting better psychological well-being and job engagement [16]. Finally, by exploring mastery climate as a moderating factor, this research underscores the significance of organizational climate in enhancing the effectiveness of DHRM practices. Specifically, it demonstrates how cultivating a mastery-oriented work environment within healthcare organizations can amplify the positive impact of DHRM, further supporting nurses' well-being and reducing feelings of organizational dehumanization.

## 2. Background

### 2.1. Theoretical Framework

SDT, developed by Ryan and Deci [18], is a widely recognized framework for understanding human motivation. It posits that individuals are most motivated and perform at their best when three basic psychological needs are fulfilled: autonomy, competence, and relatedness [20]. Specifically, autonomy reflects the need to feel in control of one's actions and decisions, competence refers to the need to feel effective and capable in one's tasks, and relatedness pertains to the need for meaningful connections and a sense of belonging with others [20, 27]. According to SDT, the fulfillment of these needs is crucial not only for fostering intrinsic motivation and well-being but also for enhancing overall performance and job satisfaction [28]. In the nursing profession, where practitioners face high emotional demands and heavy workloads, supporting these psychological needs is particularly critical. When nurses perceive greater autonomy in patient care decisions, feel competent in their clinical tasks, and experience meaningful connections with colleagues and patients, they are more likely to find purpose in their work [19]. Importantly, SDT suggests that the fulfillment of needs translates into a more explicit and observable psychological state—work meaningfulness—which captures whether employees perceive their work as valuable, purposeful, and aligned with their professional identity. Work meaningfulness thus serves as the proximal psychological mechanism through which basic need satisfaction exerts its effects, and it plays a pivotal role in counteracting experiences of objectification. Such a sense of meaningfulness is essential for mitigating negative outcomes like organizational dehumanization, which can influence nurses' well-being and the quality of care delivered [38].

Building on this foundation, the current study examines how DHRM practices can fulfill these psychological needs to enhance nurses' work meaningfulness and reduce organizational dehumanization. In nursing management, DHRM initiatives such as tailored career development plans, continuous professional training, and constructive performance feedback not only equip nurses with essential clinical competencies but also empower them to take ownership of their professional growth. These practices directly support autonomy, competence, and relatedness by recognizing nurses as valued contributors to healthcare delivery. By highlighting work meaningfulness as the central outcome of these processes, our study underscores how DHRM can transform need satisfaction into a concrete psychological resource that protects nurses from organizational dehumanization.

Furthermore, fostering a mastery climate within healthcare settings cultivates an environment that encourages nurses to embrace challenges, engage in collaborative learning, and pursue continuous improvement [24]. This growth-oriented atmosphere helps nurses feel more competent and autonomous in their roles while strengthening their relational ties with team members. By reinforcing these psychological needs, a mastery-oriented climate enhances the effectiveness of DHRM in fostering nurses' sense of work meaningfulness. Consequently, it plays a vital role in mitigating the detrimental effects of organizational dehumanization, ultimately contributing to better nurse retention, job satisfaction, and quality of patient care in healthcare organizations.

### 2.2. DHRM and Nurses' Work Meaningfulness

Work meaningfulness is defined as the degree to which individuals perceive their work as purposeful, significant, and personally fulfilling [29]. It is a critical psychological construct that drives intrinsic motivation and promotes positive work outcomes, such as engagement and well-being [30, 31]. According to SDT, individuals are more likely to experience a sense of work meaningfulness when their core psychological needs—namely, autonomy, competence, and relatedness—are satisfied [28, 32]. DHRM practices, which encompass career development opportunities, training, and performance appraisal, are uniquely positioned to fulfill these needs and enhance nurses' sense of meaningful work [16].

One important aspect of DHRM is career development, which provides nurses with structured opportunities to pursue long-term professional growth and advancement [16, 33]. By enabling them to set career goals and engage in self-directed development activities, career development supports autonomy and competence. This, in turn, strengthens nurses' sense of purpose and helps them perceive their work as more meaningful [28]. Another central dimension is training opportunities, which enable nurses to acquire new skills and improve existing ones, thereby fostering competence [17]. When training is conducted in collaborative and supportive settings, it also promotes relatedness by creating shared learning experiences. These outcomes enhance nurses' belief in their capabilities and their sense of connection to colleagues, reinforcing the meaningfulness of their work [34]. Finally, developmental performance feedback provides constructive guidance that helps nurses improve and grow [35, 36]. When feedback emphasizes learning and development rather than surveillance or control, it strengthens competence and relatedness. Nurses feel that their contributions are recognized and that they are progressing in their roles, which deepens their sense of value and significance at work [28].

Taken together, these three bundles of DHRM practices satisfy nurses' psychological needs, thereby cultivating a stronger sense of work meaningfulness. This heightened meaningfulness serves as the proximal mechanism through which DHRM reduces perceptions of organizational dehumanization.

H1: DHRM will be positively related to nurses' work meaningfulness.

### 2.3. The Mediator Role of Work Meaningfulness Between DHRM and Organizational Dehumanization

Organizational dehumanization refers to “the experience of an employee who feels objected by his or her organization, denied personal subjectivity, and made to feel like a tool or instrument for the organization's ends [8, 9, 37].” This perception typically arises in work environments characterized by excessive control, lack of recognition, or purely transactional relationships between nurses and the hospital [12]. When nurses feel dehumanized, they may experience diminished well-being, reduced engagement, and a sense of alienation from their work [12, 38, 39]. In line with our research question, we highlight work meaningfulness as the key mediator linking DHRM to organizational dehumanization. Experiencing work as meaningful directly counters feelings of objectification, making it more conceptually relevant than a general state of needs fulfillment. Moreover, work meaningfulness serves as a proximal and observable psychological state that reflects the satisfaction of basic needs and provides a clear explanation of how DHRM practices reduce dehumanization.

First, work meaningfulness fosters a sense of personal significance, allowing nurses to perceive their efforts and contributions as valuable and impactful [31]. When nurses view their work as meaningful, they are less likely to feel reduced to mere tools serving hospital's objectives. This sense of significance helps them maintain their dignity and intrinsic worth, countering the core elements of organizational dehumanization [40]. Second, work meaningfulness strengthens nurses' relational ties to the organization by creating a shared sense of purpose and alignment with organizational goals [37]. Nurses who find meaning in their work are more likely to perceive the organization as valuing their individuality and fostering mutual respect [41]. These positive relational dynamics mitigate feelings of detachment that are characteristic of organizational dehumanization. Finally, work meaningfulness provides a psychological reframing of nurses' roles within the hospital [41]. Rather than viewing themselves as replaceable or interchangeable, nurses who find meaning in their work interpret their roles as integral to the hospital's success. This reframing not only diminishes perceptions of organizational dehumanization but also promotes a more engaged and empowered workforce.

In sum, DHRM plays a key role in enhancing nurses' sense of work meaningfulness by fulfilling their three core psychological needs. When nurses feel they have the freedom to make decisions (autonomy), are able to grow and apply their skills (competence), and experience positive social connections with others (relatedness), they are more likely to find deeper meaning in their work [34]. Work meaningfulness thus represents the explicit psychological state that translates the satisfaction of basic needs into a deeper connection with one's work. Compared with the more general notion of needs fulfillment, work meaningfulness provides a clearer and more observable mechanism for explaining how organizational practices shape employees' daily experiences. This heightened sense of work meaningfulness acts as a buffer, reducing the likelihood that nurses will perceive their organization as dehumanizing [38]. By experiencing their work as meaningful, nurses are less likely to feel treated as mere instruments and more likely to view themselves as valued contributors to healthcare delivery. Therefore, we propose the following hypothesis.

H2: DHRM will have an indirect, negative effect on organizational dehumanization through nurses' work meaningfulness.

### 2.4. The Moderator Role of Mastery Climate Between DHRM and Work Meaningfulness

In the workplace, employees' psychological experiences are shaped not only by formal HR practices but also by the broader organizational climate. While DHRM practices provide structural support for nurses' growth through opportunities for development, constructive feedback, and skill enhancement, the surrounding climate determines how these practices are interpreted and enacted. In particular, a mastery climate—a growth-oriented environment that emphasizes effort, learning, and collaboration [22]—can serve as a boundary condition that amplifies the effectiveness of DHRM. When such a climate is present, nurses are more likely to view DHRM practices as genuine support for their development, thereby strengthening their sense of autonomy, competence, and relatedness. Conversely, in the absence of a mastery climate, the motivational signals of DHRM may be weakened or even undermined. Thus, mastery climate is expected to magnify the positive relationship between DHRM and work meaningfulness.

A mastery climate, which emphasizes effort, collaboration, learning, and self-development [43, 44], is characterized by valuing progress over competition, encouraging mutual support, and recognizing individual improvement. Unlike a performance climate, which stresses comparison and evaluation, a mastery climate fosters psychological safety and authentic growth. Such an environment is particularly critical in nursing, where teamwork, continuous learning, and professional development are essential for both effectiveness and well-being [22]. Within this context, mastery climate plays a crucial moderating role in enhancing the effectiveness of DHRM. By creating a supportive atmosphere where nurses are encouraged to take initiative and pursue personal as well as professional growth, a mastery climate acts as an amplifier of formal HRM practices. It strengthens the positive influence of DHRM on nurses' sense of competence, autonomy, and relatedness, making these practices more meaningful and impactful. This, in turn, not only heightens their work meaningfulness but also deepens their psychological connection to the hospital, thereby reducing the likelihood of experiencing organizational dehumanization.

First, a mastery climate promotes autonomy by creating an environment that encourages self-directed learning, initiative, and personal growth [43]. When such a climate coexists with DHRM practices (e.g., career development opportunities), nurses are more likely to interpret these practices as genuine resources for their self-development, thereby feeling a stronger sense of control over their professional growth and experiencing greater work meaningfulness. Second, a mastery climate strengthens competence by emphasizing continuous learning, effort, and skill development [42, 49]. In combination with DHRM practices such as training and constructive feedback, this climate reinforces nurses' confidence in their abilities, making them feel more capable and effective in their roles. This synergy not only enhances competence but also deepens their sense of purpose and value at work. Finally, a mastery climate nurtures relatedness by fostering collaboration, mutual support, and a sense of community among nurses [25]. When DHRM initiatives such as mentorship programs or team-based projects are implemented in such an environment, nurses are more likely to experience authentic connections with peers and the hospital, thereby amplifying the relational benefits of DHRM and strengthening their sense of work meaningfulness.

In sum, it is the interaction between mastery climate and DHRM practices that creates the most powerful effects: While DHRM provides the formal structures to support growth, mastery climate amplifies their motivational impact, leading to a deeper sense of work meaningfulness.

H3: Mastery climate will strengthen the positive relationship between DHRM and nurses' work meaningfulness, such that the relationship will be stronger when mastery climate is higher than when it is lower.

### 2.5. The Integrative Moderated Mediation Model

In summary, a mastery climate plays a crucial moderating role in strengthening the impact of DHRM on nurses' work meaningfulness, which in turn reduces perceptions of organizational dehumanization. A mastery climate—characterized by a focus on growth, learning, collaboration, and skill development—creates the conditions under which DHRM practices are most effective. For example, when nurses are encouraged to set personal development goals and engage in continuous skill building, DHRM initiatives such as personalized career development plans and constructive feedback are more likely to foster a strong sense of purpose and competence.

A mastery climate, characterized by an emphasis on learning, effort, collaboration, and self-improvement, acts as an amplifier of HRM practices [30, 31]. It conveys to nurses that the organization values long-term development and collective growth, thereby enhancing the credibility and motivational impact of DHRM [45]. In a supportive and mastery-oriented climate, nurses are more likely to feel valued and respected, which lessens the emotional distance that can lead to disengagement [42]. Nurses are thus more likely to interpret DHRM initiatives as authentic resources for their development, which strengthens their sense of autonomy, competence, and relatedness. Through this interaction, nurses experience a heightened sense of work meaningfulness. Work meaningfulness functions as the proximal psychological mechanism that translates structural opportunities and climate signals into personal experiences of value and purpose. When nurses feel that their work is significant and aligned with their identity, they are less likely to view themselves as replaceable tools. In this way, meaningful work directly counters feelings of objectification, thereby reducing perceptions of organizational dehumanization.

In contrast, when mastery climate is weak, the developmental signals provided by HRM may be diluted or even questioned. Nurses might then perceive training and feedback as compliance-oriented or instrumental, which limits their potential to generate meaningfulness and, consequently, their ability to buffer against organizational dehumanization. Thus, neither DHRM nor mastery climate alone is sufficient; rather, it is their interaction through work meaningfulness that best explains how organizations can mitigate organizational dehumanization in high-demand settings such as healthcare.

H4: Mastery climate will strengthen the negative indirect effect of DHRM on organizational dehumanization through work meaningfulness, such that the negative indirect effect will be stronger when mastery climate is higher than when it is lower.

## 3. Methods

### 3.1. Design

We conducted this research and tested our hypotheses in a hospital based in Beijing, China. The choice of the healthcare sector is particularly relevant, given the industry's high work intensity, fast-paced environment, and continuous demand for medical advancements. These characteristics make it an ideal context for exploring nurses' perceptions of organizational dehumanization, which can be more pronounced in healthcare settings with high workloads, long shifts, and rapidly evolving medical technologies. The hospital's emphasis on patient care and performance outcomes provides a unique setting for studying how organizational practices and environmental factors influence nurses' experiences, job satisfaction, and overall well-being.

### 3.2. Procedure and Participants

With the assistance of two human resource managers from the hospital, we distributed the surveys across three rounds to a sample of frontline nurses via email and WeChat. Participants were fully informed about the purpose of the study and assured that their responses would remain confidential and be used solely for academic purposes. Additionally, they were reminded that participation was entirely voluntary, and they could withdraw at any time without consequence. To encourage participation and thank respondents for their time, each participant was compensated with 15 RMB (approximately 2 USD) upon completion of the survey. To ensure data quality, each wave of the survey also included an attention check item (e.g., instructing participants to select a specific response option), and responses failing this check were excluded from the analyses.

To minimize common method bias (CMB) [46], we employed a three-wave time-lagged design, collecting data from the same participants at three points in time with approximately 1-month intervals between each wave. To link responses across the three waves, participants were asked to create self-generated anonymous codes (e.g., a combination of letters or numbers only known to themselves). This procedure enabled us to match data across waves while ensuring that no identifying information (such as names or employee IDs) was collected.

Specifically, demographic variables and nurses' perceptions of DHRM and mastery climate were measured at T1, work meaningfulness at T2, and organizational dehumanization at T3. Of the 340 nurses invited, 328 completed the survey at T1, 319 at T2, and 311 at T3, yielding a final matched sample of 311. The proportion of missing data was low (3.5% at T1, 2.7% at T2, and 2.5% at T3), and missing cases were handled using listwise deletion. Following Goodman and Blum's [47] recommendations, we conducted independent-samples *t*-tests to compare participants who dropped out after T1 with those who remained in the study on the variables measured at baseline (DHRM, mastery climate, and the control variables). No significant differences were found, suggesting that attrition did not bias our results. Among 311 nurses, 99.4% of the participants were female, the average age was 33.32 years, the average tenure in the organization was 5.30 years, and 58.2% held a bachelor's degree, while 7.4% possessed a master's degree. They are distributed across various departments, including common ones such as internal medicine, surgery, pediatrics, and emergency care.

### 3.3. Measures

In this study, participants were asked to respond to the measures using a six-point Likert scale, ranging from “*strongly disagree*” (1) to “*strongly agree*” (6). All scales originally developed in English were translated into Chinese using the standard back-translation procedure [47]. Specifically, two bilingual researchers independently translated the items into Chinese, and a third bilingual researcher who was blinded to the original scales back-translated them into English. Discrepancies between the original and back-translated versions were discussed and resolved to ensure semantic and cultural equivalence.

#### 3.3.1. DHRM

We utilized Kuvaas's [16] scale to measure DHRM. A total of 21 items were used to capture nurses' perceptions of DHRM practices, covering three core dimensions: career development, training opportunities, and developmental performance feedback. A sample item was “My hospital really cares about my career opportunities.” Cronbach's alpha was 0.92. As highlighted in previous research, employees within the same organization may have varied perceptions of HRM, and it is these subjective perceptions of HRM that have a more direct impact on their attitudes and states. Thus, we chose to measure nurses' perceptions of DHRM. Scores for each bundle were computed by averaging its respective items, and these bundle scores were used as indicators of the overall DHRM construct.

#### 3.3.2. Mastery Climate

Mastery climate was measured using the six-item scale developed by Nerstad et al. [49]. A sample item was “In my hospital, each individual's learning and development is emphasized.” Cronbach's alpha for this scale was 0.95.

#### 3.3.3. Work Meaningfulness

We assessed work meaningfulness with the three-item scale developed by Spreitzer [50]. This measure has been the most widely used scale in work meaningfulness literature. One sample item was “My job activities are personally meaningful to me.” Cronbach's alpha was 0.93.

#### 3.3.4. Organizational Dehumanization

The perception of organizational dehumanization was measured using a scale consisting of 11 items developed by Caesens et al. [6]. A sample item was “My hospital treats me as if I were a robot.” Cronbach's alpha was 0.96.

#### 3.3.5. Control Variables

We controlled for nurses' age, gender, education level, and organizational tenure, as these sociodemographic characteristics may systematically influence employees' work attitudes and perceptions [51]. Prior research has shown that age is associated with differences in job attitudes and adaptability [52], gender may shape work role expectations [53], and education level can influence skill sets and career expectations [54]. Controlling for these variables therefore helps reduce the risk of alternative explanations and ensures a more conservative test of our hypotheses. Age and tenure were treated as continuous variables; gender was coded as 1 = female and 2 = male; and education was coded as 1 = high school or below, 2 = associate degree, 3 = bachelor's degree, and 4 = master's degree or above. The results showed that none of the control variables had a significant effect on organizational dehumanization. Nevertheless, we retained them in our model to provide a more conservative test [51]. 1

### 3.4. Discriminant Validity

Prior to hypothesis testing, we performed a confirmatory factor analysis to test the distinctiveness of the key variables. To minimize the number of parameters in the model and retain a fair degree of freedom, we employed an item parceling strategy, which is widely recommended when constructs are measured with many items and the sample size is relatively modest. Parcels in structural equation modeling (SEM) help maintain a manageable indicator-to-sample size ratio [55] and provide an adequate representation of latent constructs [56]. Specifically, following Little et al.'s [57] recommendations, we applied theory-guided parceling and conducted exploratory factor analysis to aid our parceling decisions. In specific, we created three parcels for DHRM based on its three practices, six parcels for organizational dehumanization by pairing its highest and lowest load items. The results in Table 1 show that the theorized four-factor model (*χ*^2^ (129) = 410.80, CFI = 0.95, TLI = 0.94, and RMSEA = 0.08) provides a good fit with the data and outperforms alternative models. Hence, all of the major constructs in this study have good discriminant validity.

Although we collected data from three time points, all of the variables were self-reported by nurses. This may have induced CMB. Hence, following the guidelines of Podsakoff et al. [58], we conducted two tests to determine whether CMB influenced this study. First, we conducted our analyses by using the single-factor method. As shown in Table 1, the single-factor model fit poorly with the data (*χ*^2^ (135) = 3834.04, CFI = 0.37, TLI = 0.28, and RMSEA = 0.30). We also used the unmeasured latent method to control for common method variance. In this approach, an additional latent method factor is included in the confirmatory factor analysis model, with each item specified to load on both its theoretical construct and the unmeasured latent method factor. By comparing the fit indices of the baseline model with and without the latent method factor, it is possible to estimate the extent of common method variance. In our study, the inclusion of the latent method factor did not significantly improve model fit (*χ*^2^ (128) = 614.60, CFI = 0.92, TLI = 0.90, and RMSEA = 0.08), indicating that CMB had a minimal impact on our findings.

## 4. Results

### 4.1. Descriptive Statistics and Correlations

Table 2 displays the means, standard deviations, and correlations among the variables. Based on the findings, the DHRM was positively related to work meaningfulness (*r* = 0.29, *p*  <  0.01) and negatively related to organizational dehumanization (*r* = −0.25, *p*  <  0.01). Mastery climate was positively related to work meaningfulness (*r* = 0.23, *p*  <  0.01) and negatively related to organizational dehumanization (*r* = −0.12, *p*  <  0.05). These findings provide preliminary support for our hypotheses.

### 4.2. Hypothesis Testing

We tested our hypotheses using SEM in Mplus 8.3 [59]. To estimate the latent interaction between DHRM and mastery climate, we employed the latent moderated structural equations (LMS) approach with the XWITH command [60]. Because conventional fit indices (e.g., *χ*^2^, CFI, TLI, RMSEA) are not available in LMS, we followed recent recommendations [61] and compared the Akaike information criterion (AIC) between the models with and without the latent interaction term. The model including the interaction showed a smaller AIC value (AIC = 10,665.75) than without the latent interaction term (AIC = 10,667.19), indicating a better fit and a greater likelihood of replication.

**Figure 2:**
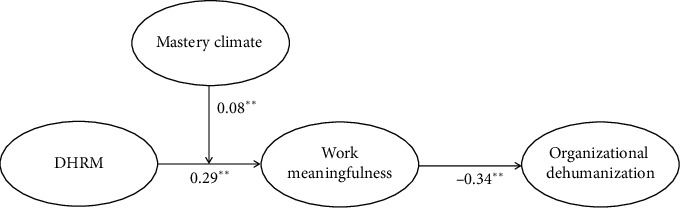
Results of structural modeling. Note: ^∗∗^*p*  <  0.01; *p*  <  0.05. All path coefficients are unstandardized. In our analyses, gender and age showed marginally significant effects on work meaningfulness. The results for control variables have been excluded from the figure for ease of presentation.

Figure 2 presents the results of our hypothesis testing. Hypothesis 1 stated the relationship between DHRM and nurses' work meaningfulness. As shown in Figure 2, after controlling for nurses' age, gender, education, and organizational tenure, DHRM had a positive effect on nurses' work meaningfulness (*b* = 0.29, *p*  <  0.01). Thus, H1 was supported. Regarding H2, the results showed that nurses' work meaningfulness is significantly negatively related to organizational dehumanization (*b* = −0.34, *p*  <  0.01). Moreover, the 95% CI for the indirect effect did not include zero (estimate = −0.10, 95% CI [−0.15, −0.04]). Thus, H2 was supported.

**Figure 3:**
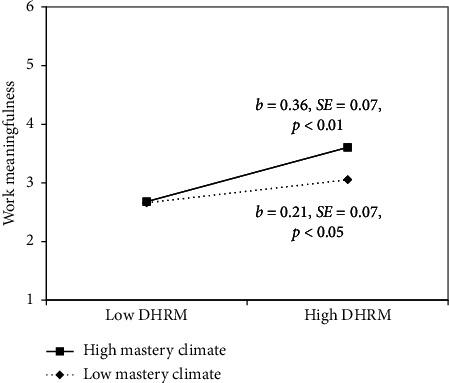
The moderating role of mastery climate between DHRM and work meaningfulness.

H3 posited that mastery climate moderates the relationship between DHRM and nurses' work meaningfulness. As shown in Figure 2, the interaction term of DHRM and mastery climate was positive and significant (*b* = 0.08, *p*  <  0.01). To unravel this significant interaction, we conducted a simple slope analysis, examining conditional effects at −1 SD/+1 SD levels of mastery climate. As presented in Figure 3, when the mastery climate was high, DHRM had a stronger positive relationship with nurses' work meaningfulness (*b* = 0.36, *p*  <  0.01) than when it was low (*b* = 0.21, *p*  <  0.05). The results suggested that the mastery climate strengthened the relationship between DHRM and nurses' work meaningfulness, as predicted by H3.

We further tested the moderated mediation effects, which were posited by H4 that the indirect effect of DHRM on organizational dehumanization through work meaningfulness is moderated by mastery climate. Specifically, we tested the conditional indirect effect at two levels of mastery climate (−1 SD/+1 SD). Specifically, the indirect effect of DHRM on organizational dehumanization through work meaningfulness was more negative when mastery climate was higher (estimate = −0.12, 95% CI [-0.19, −0.05]) than when mastery climate was lower (estimate = −0.07, 95% CI [-0.12, −0.02). The difference between these two indirect effects was significant (difference estimate = −0.05, 95% CI [-0.10, −0.003]). The results support Hypothesis 4.

## 5. Discussion

This study applied SDT to examine how DHRM practices reduce perceptions of organizational dehumanization. Using a sample of 311 nurses from Chinese hospitals, our findings indicated that DHRM was positively associated with nurses' work meaningfulness, which was in turn linked to lower perceptions of organizational dehumanization. Furthermore, the study found that a mastery climate moderated the relationship between DHRM and work meaningfulness. Specifically, nurses who perceived a stronger mastery climate reported a greater sense of work meaningfulness, which further mitigated the experience of organizational dehumanization. In the following section, we discuss the theoretical and practical implications of these findings.

### 5.1. Theoretical Implications

First, by exploring the relationship between DHRM and organizational dehumanization, this study addresses an important gap in the literature by exploring the strategies that help mitigate organizational dehumanization. While previous research has identified the negative consequences and triggering factors of organizational dehumanization, much less attention has been paid to how to reduce this harmful perception [7–11]. Only one study has explored the role of organizational support in alleviating organizational dehumanization [6], which is insightful but still insufficient. This is because organizational support focuses mainly on the emotional and relational aspects of the workplace, while other organizational factors, such as HR practices, may also play a critical role in shaping employees' perceptions and experiences. Thus, this study adopts an organizational practices perspective, demonstrating how DHRM, by fulfilling nurses' basic psychological needs, subsequently reduces perceptions of organizational dehumanization. Our findings provide a systematic, institutional-level explanation of how and why DHRM practices can mitigate organizational dehumanization, thereby enriching the literature on organizational dehumanization and expanding the scope of future research on this topic.

Second, by revealing the mediating role of work meaningfulness, this study uncovers the psychological process through which organizational dehumanization can be alleviated. As mentioned earlier, research on factors that mitigate organizational dehumanization, especially the psychological mechanisms at play, remains scarce [12]. This gap complicates the efforts to address the root causes of organizational dehumanization in the workplace. Our study addresses this gap by highlighting work meaningfulness as a core psychological mechanism. We demonstrate how DHRM practices, by fulfilling nurses' basic psychological needs, foster a stronger sense of purpose and meaning in their work, which directly counters the perception of organizational dehumanization [59]. Additionally, by clarifying the causal link between DHRM and work meaningfulness, this research offers a deeper understanding of how organizational practices can address the psychological roots of dehumanization. This, in turn, offers valuable insights for both advancing theoretical understanding and informing practical interventions within the HRM literature.

Third, this study's focus on mastery climate as a moderator adds a valuable dimension to the HRM literature by highlighting the overlooked role of the work environment. While much research has focused on the direct effects of DHRM practices on employee outcomes [16, 17], the impact of organizational climate—especially a mastery-oriented one—has been largely underexplored in the existing DHRM literature. This study demonstrates that a positive mastery climate can strengthen the effects of DHRM on work meaningfulness, offering new insights into how organization's climate can enhance the effectiveness of HR strategies. Besides, our results demonstrated that a mastery climate, which fosters learning and growth, not only strengthens the relationship between DHRM and work meaningfulness but also has a negative correlation with organizational dehumanization (*r* = −0.12, *p*  <  0.05). This also implies that a mastery climate has the potential to effectively mitigate employees' perceptions of organizational dehumanization, a direction that future research could explore and test further. Therefore, by addressing both organizational practices and climate factors, this study provides a more comprehensive understanding of how to mitigate organizational dehumanization.

### 5.2. Practical Implications

Our findings offer several important implications for hospital management. First, the results suggest that hospitals should implement DHRM practices that prioritize individualized training programs, career development planning, and constructive performance appraisal. By focusing on developmental rather than punitive approaches, hospitals can create a supportive environment in which nurses feel respected and motivated, thereby reducing experiences of objectification [1]. Second, the study highlights the central role of work meaningfulness in mitigating organizational dehumanization. Hospitals can enhance nurses' sense of meaning by clarifying the link between daily tasks and the broader mission of patient care, granting autonomy in decision making, and providing opportunities for employees to take ownership of projects. Third, our findings underscore the importance of cultivating a mastery-oriented climate. Leaders can foster such a climate by promoting collaboration, supporting team-based initiatives, encouraging knowledge sharing, and rewarding continuous improvement, all of which amplify the positive impact of DHRM and foster greater work meaningfulness.

Beyond organizational practice, this study also provides insights for public policymakers. At the policy level, governments and health authorities can incorporate DHRM principles into healthcare workforce standards to ensure that professional development opportunities and supportive appraisal systems are systematically available across institutions. Policymakers may also design funding schemes and incentive programs to encourage hospitals to build mastery-oriented and supportive climates. By implementing such measures, policymakers can reduce nurses' experiences of organizational dehumanization on a larger scale, improve their well-being, and ultimately sustain the quality and resilience of healthcare delivery.

### 5.3. Limitations and Directions for Future Research

Despite the valuable contributions of this study, several limitations must be acknowledged, and future research can expand on these areas. First, because all data were collected through self-reported surveys, the responses may have been influenced by social desirability bias, particularly for sensitive constructs such as organizational dehumanization. Although our three-wave time-lagged design helped reduce CMB and establish temporal ordering, it does not constitute a full longitudinal panel design that would allow dynamic changes and reciprocal relationships among DHRM, work meaningfulness, and organizational dehumanization to be captured. Due to hospital regulations and the heavy workload of nurses, repeated measurement of the same constructs over time was not feasible in the present study. Future research should therefore employ more rigorous designs—such as multisource data, cross-lagged panel or growth models, or even experimental approaches—to better address social desirability concerns, strengthen causal inference, and reveal the temporal dynamics underlying these relationships.

Second, our sample was predominantly female, reflecting the gender distribution of the nursing profession in China. While this enhances ecological validity, it also constrains the generalizability of the findings to more gender-balanced samples or to other occupational groups. Future research should test whether the observed mechanisms hold among male nurses or in different professional contexts where gender roles may shape HRM perceptions differently.

Lastly, the study was conducted within a single cultural and institutional context (Chinese hospitals). Cultural norms and institutional arrangements may influence how DHRM practices, mastery climate, and work meaningfulness are perceived [48]. Consequently, the generalizability of our findings beyond this context is limited. Future research should therefore examine these relationships across diverse cultural and institutional settings to test the robustness and boundary conditions of our model.

## 6. Conclusion

As organizational dehumanization becomes an increasingly discussed issue in hospital settings, scholars have examined its negative effects and potential antecedents. However, research has not sufficiently addressed strategies that might help mitigate this phenomenon. Drawing on SDT, this study explores the potential role of DHRM in relation to nurses' experiences of organizational dehumanization by examining its association with work meaningfulness. Specifically, DHRM may support nurses' core psychological needs for autonomy, competence, and relatedness, which are linked to a stronger sense of meaningful work. In addition, our findings suggest that a mastery climate may shape how nurses experience work meaningfulness, such that a growth-oriented environment appears to strengthen the positive association between DHRM and meaningful work. This study extends research on organizational dehumanization by highlighting possible organizational practices that are connected to more positive experiences, and it contributes to the DHRM literature by offering further insights into its potential effectiveness in healthcare contexts.

## Figures and Tables

**Table 1:** Comparison of measurement models.

Models	*χ* ^2^	*df*	Δ*χ*^2^ (Δ*df*)	CFI	TLI	RMSEA
Model 1: DHRM, MC, WM, OD	410.80	129	—	0.95	0.94	0.08
Model 2: DHRM + MC, WM, OD	821.17	132	410.37(3)	0.88	0.86	0.13
Model 3: DHRM, MC + WM, OD	1247.71	132	836.91(3)	0.81	0.78	0.17
Model 4: DHRM + MC + WM, OD	1641.42	134	1230.62(5)	0.74	0.71	0.19
Model 5: DHRM + MC + WM + OD	3834.04	135	3423.24(6)	0.37	0.28	0.30

*Note:* The alternative models were compared with Model 1.

Abbreviations: DHRM, developmental human resource management; MC, mastery climate; OD, organizational dehumanization; WM, work meaningfulness.

*p*  <  0.01.

**Table 2:** Means, standard deviations, and correlations among variables (*N* = 311).

Variables	*M*	*SD*	1	2	3	4	5	6	7
1. Age	33.32	5.32							
2. Gender	1.01	0.08	0.03						
3. Education	2.68	0.68	−0.20^∗∗^	−0.02					
4. Organizational tenure	5.30	2.86	0.42^∗∗^	0.05	−0.32^∗∗^				
5. DHRM	4.54	0.76	0.06	0.09	−0.04	−0.002			
6. Mastery climate	4.57	1.02	−0.004	0.08	0.08	−0.03	0.63^∗∗^		
7. Work meaningfulness	4.67	0.10	0.09	0.07	−0.06	0.01	0.29^∗∗^	0.23^∗∗^	
8. Organizational dehumanization	2.06	0.75	−0.12^∗^	−0.05	−0.04	−0.04	−0.25^∗∗^	−0.12^∗^	−0.41^∗∗^

^∗∗^ *p*  <  0.01; ^∗^*p*  <  0.05.

## Data Availability

The data that support the findings of this study are available from the corresponding author upon reasonable request.

## Nomenclature

DHRM: Developmental human resource management

## Endnotes

^1^Supplementary analyses estimated models without control variables, and the results remained consistent with those reported in the main analyses. Full results are available from the authors upon request.
